# The Precarious Health of Young Mexican American Men in South Texas, Cameron County Hispanic Cohort, 2004–2015

**DOI:** 10.5888/pcd13.160020

**Published:** 2016-08-25

**Authors:** Gordon P. Watt, Kristina P. Vatcheva, Derek M. Griffith, Belinda M. Reininger, Laura Beretta, Michael B. Fallon, Joseph B. McCormick, Susan P. Fisher-Hoch

**Affiliations:** Author Affiliations: Kristina P. Vatcheva, Belinda M. Reininger, Joseph B. McCormick, Susan P. Fisher-Hoch, University of Texas School of Public Health, Brownsville Regional Campus, Brownsville, Texas; Derek M. Griffith, Institute for Research on Men’s Health, Vanderbilt University, Nashville, Tennessee; Laura Beretta, Department of Molecular and Cellular Oncology, The University of Texas, MD Anderson Cancer Center, Houston, Texas; Michael B. Fallon, Division of Gastroenterology, Hepatology, and Nutrition, The University of Texas Health Science Center at Houston Medical School, Houston, Texas.

## Abstract

**Introduction:**

Hispanic men have higher rates of illness and death from various chronic conditions than do non-Hispanic men. We aimed to characterize the health of Mexican American men living on the US–Mexico border in South Texas and elucidate indications of chronic disease in young men.

**Methods:**

We sampled all male participants from the Cameron County Hispanic Cohort, an ongoing population-based cohort of Mexican Americans in Brownsville, Texas. We calculated descriptive statistics and stratified the sample into 3 age groups to estimate the prevalence of sociodemographic, behavioral, and clinical factors by age group and evaluated differences between age groups.

**Results:**

Obesity prevalence was approximately 50% across all age groups (*P* = .83). Diabetes prevalence was high overall (26.8%), and 16.9% (95% confidence interval [CI], 10.1%–23.8%) of men younger than 35 had diabetes. More than 70% of these young men had elevated liver enzymes, and mean values of aspartate aminotransferase were significantly higher in younger men (45.0 u/L; 95% CI, 39.5–50.6 u/L) than in both older age groups. Less than 20% of young men had any form of health insurance. Current smoking was higher in young men than in men in the other groups, and the rate was higher than the national prevalence of current smoking among Hispanic men.

**Conclusions:**

We suggest a need for obesity and diabetes prevention programs and smoking cessation programs for men in this region. Opportunities exist to expand current intervention programs and tailor them to better reach this vulnerable population of young Hispanic men. Elevated liver enzymes in men younger than 35 suggest a substantial burden of liver abnormalities, a finding that warrants further study.

## Introduction

Hispanic men comprise a rapidly growing demographic of the United States, yet the research literature on Hispanic men’s health is sparse. Studies indicate that Hispanics in the United States face many health inequities, ranging from lack of access to care to higher rates of infectious and chronic diseases (1–4). However, few studies have addressed health inequalities in Hispanic men (3,4). Despite having a longer life expectancy than non-Hispanic white men (5), Hispanic men have higher mortality rates than do non-Hispanic men for chronic conditions such as type 2 diabetes, end-stage renal disease, colorectal cancer (6), and liver disease (7). Research suggests that a lack of access to care contributes to this increased risk of death from chronic conditions in Hispanic men (8).

Hispanic men are an epidemiologically heterogeneous group. Improvements in Hispanic health surveillance data (9) and data from the Hispanic Community Health Study/Study of Latinos (HCHS/SOL) and others indicate disparate health outcomes in Hispanics by ethno-regional subgroup (3,10,11). However, to date, studies that examined the health of Hispanic ethnic subgroups in the United States neglect men’s health; of the 37 publications that emerged from the HCHS/SOL, none specifically studied men’s health (12). As an additional illustration of the dearth of health research in US Hispanic men, a 2016 MeSH term search of “men’s health” and “Hispanic Americans or Mexican Americans” on Ovid Medline yielded only 20 relevant articles since 1946.

This study describes the health characteristics of Mexican American men on the southern Texas–Mexico border in Brownsville, Texas. This area is resource-poor, among the poorest cities in the United States (13). With a population of primarily Mexican descent (93.2% Hispanic, of whom 86.2% are of Mexican descent), data from this ethnically homogenous population of men are ideal for comparing with those of nationwide surveys of Mexican American men’s health (such as the National Health and Nutrition Examination Survey and HCHS/SOL). We have shown that this population has high rates of obesity, insulin resistance, diabetes, liver disease, and subclinical atherosclerosis, with significant differences by sex (14–19). Additionally, in reviewing the data from the Cameron County Hispanic Cohort (CCHC), a population-based cohort study of the region, we observed high rates of obesity in younger men (younger than 35 years). The purpose of this study is to further characterize men’s health by age group to assess the burden of chronic disease in younger men. Age is a relevant trait for risk stratification and intervention, and a precise characterization of the population is fundamental to any further work in Mexican American men.

## Methods

We conducted a cross-sectional analysis of 945 men in the CCHC from Brownsville, Texas, recruited from 2004 through 2015. The CCHC is a representative sample of the Mexican American population in South Texas, selected by a 2-stage methodology using US Census data; socioeconomic quartiles are the sampling strata, and census blocks are the randomized sampling units. We invited all members (aged 18 years or older) of all households in the selected blocks to participate in the CCHC. Participants gave informed consent and then came to the local clinical research unit where extensive sociodemographic, clinical, and laboratory data were collected. We have detailed these protocols previously (14,15). Trained bilingual health workers conducted a clinical exam and health interview in the participants’ language of preference (English or Spanish). Participants arrived fasting for 10 hours so that blood samples could be obtained for a comprehensive metabolic panel, complete blood count with differential, and hemoglobin A1c (HbA1c) measurements. The study team measured anthropometrics and blood pressure and asked participants a range of demographic, clinical, health care use, and health behavior questions.

### Definitions

The definition of elevated liver function tests (LFTs) was alanine transaminase (ALT) levels greater than 40 u/L and/or aspartate aminotransferase (AST) levels greater than 37 u/L. We used the 2010 American Diabetes Association diagnostic criteria for diabetes mellitus (DM) (20). A body mass index (BMI) of more than 30 kg/m^2^ indicated obesity. We defined hypertension as mean systolic blood pressure (SBP) at or above 130 mm Hg or a mean diastolic blood pressure (DPB) at or above 85 mm Hg, or taking antihypertensive medication. Three or more of the following constituted metabolic syndrome: hypertension, triglyceride levels over 150 mg/dL, high-density lipoprotein (HDL) cholesterol levels less than 40 mg/dL, fasting blood glucose over 100 mg/dL or taking hypoglycemic medication, or waist circumference higher than 102 cm (21). Total cholesterol of more than 200 mg/dL indicated hypercholesterolemia. A “heavy drinker” was any participant reporting drinking more than 14 alcoholic drinks per week (22). We defined history of smoking as an affirmative response to the question, “Have you ever smoked more than 100 cigarettes in your entire life?” and current smoking as an affirmative response to the question, “Do you now smoke cigarettes?” among those with a history of smoking.

### Statistical methods

The CCHC has a 2-stage population-based sampling methodology. To address possible sampling bias, we adjusted all analyses for the probability of sampling using age- and sex-adjusted sampling weights, based on the population of Brownsville, Texas. We also accounted for the potential clustering effect among participants from the same household or census block. We considered results statistically significant at *P* < .05. In descriptive analyses, we calculated unweighted frequencies and weighted proportions for categorical variables. We then stratified participants into 3 age groups (younger, aged 18–34 years; middle-aged, 35–54 years; and older, aged ≥55 years) to estimate health outcomes by age group. Weighted proportions of participants in each age group were approximately equal (Rao-Scott χ^2^ = 1.3, *P* = .53).

We used the Rao-Scott χ^2^ test to assess equality of proportions of categorical variables across age groups. For continuous variables, we used survey-weighted linear regression analysis to detect differences in mean values across age groups. For blood pressure analysis, we adjusted for current antihypertensive medication, and for analyses of triglycerides and LDL and HDL cholesterol variables, we adjusted for lipid-controlling medications. Because this population is binational, we sought to rule out confounding of place of birth on the results; using logistic and linear regression, we repeated each analysis controlling for place of birth. Where the adjustment for place of birth affected results, we have noted this effect; otherwise, crude results are presented.

In the case of a significant independent overall association of age with the variable of interest, we used multiple comparisons techniques to detect significant differences in the younger age group compared with the other age groups. For categorical associations, we used logistic regression to calculate odds ratios (ORs) for the outcome of interest in the younger age group compared with the other age groups. For significant associations with continuous variables, we applied regression models to obtain *t*-test results with Bonferroni-adjusted *P* values for multiple pairwise comparisons across age groups; we reported the difference of least square means and associated 95% confidence intervals (CIs) and *P* values. For all analyses, we used SAS version 9.4 (SAS Institute, Inc).

## Results

### Characteristics of the study population

We excluded 86 participants who were missing critical data; our final sample was 945 men from the CCHC. Mean age of participants was 44.3 years, and approximately half (50.4%) were born in Mexico (Table 1). The mean age of men born in the United States (40.4 y) was significantly lower than the mean age of men born outside the United States (48.0 y, *P* = *.*002; data not shown). Confounding by place of birth was significant for only one analysis: multiple comparisons of AST levels. The remaining results were not significantly affected when adjusting for place of birth, so crude results are presented. Most participants (63.6%) had no medical insurance (including Medicare and Medicaid), and 18.9% were unemployed (not retired) at the time of the interview. Many participants (38.5%) declined to provide income data. Approximately half of the participants had a history of smoking (54.2%), and 28.4% were current smokers. With regard to drinking habits, 7.3% reported heavy drinking, and 60.3% reported occasional alcohol consumption. We found high rates of metabolic abnormalities: 49.9% of participants were obese, 26.8% had diabetes, 44.0% met the criteria for metabolic syndrome, and 64.4% had elevated liver enzymes (Table 1).

**Table 1:** Descriptive Statistics of Men in the Cameron County Hispanic Cohort (N = 945), 2004–2015

Participant Characteristic	Value^a^
**Mean age, y (SE) (n = 945)**	44.3 (1.0)
**Mean years in Brownsville (SE) (n = 945)**	28.2 (1.9)
**Mean weight, kg (SE) (n = 945)**	90.3 (1.0)
**Mean height, cm (SE) (n = 945)**	170.7 (0.3)
**Mean waist circumference, cm (SE) (n = 945)**	105.2 (0.7)
**Mean waist-to-hip ratio (SE) (n = 945)**	1.0 (0)
**Mean body mass index, kg/m^2^ (SE) (n = 945)**	30.9 (0.3)
**Marital status (n = 944)**	
Single/never married	21.6
Married	71.5
Divorced/separated	5.9
Widowed	1.1
**Insurance^b^ (n = 944)**	
No insurance	63.6
Insurance	36.3
**Employment (n = 944)**	
Retired	15.2
Full-time	46.9
Part-time	14.7
Unemployed	18.9
Not in workforce	4.4
**Place of birth (n = 945)**	
United States	48.0
Mexico	50.4
Other	1.6
**Education level (n = 945)**	
Completed high school	58.3
Did not complete high school	41.7
**History of smoking^c^ (n = 945)**	
Yes	54.2
No	45.8
**Current smoker^d^ (n = 945)**	
Yes	28.4
No	71.6
**Drinking (n = 944)**	
Never	39.7
Sometimes	60.3
**Heavy drinking (drinks/wk) (n = 945)**	
Yes (>14)	7.3
No (≤14)	92.7
**Elevated liver enzymes^e^ (n = 945)**	
No	35.6
Yes	64.4
**Body mass index categories (kg/m^2^) (n = 945)**	
Not obese (≤ 30)	50.1
Obese (>30)	49.9
**Diabetes categories^f^ (n = 945)**	
Normal	35.9
Prediabetes	37.3
Diabetes	26.8
**Hypertriglyceridemia (mg/dL) (n = 945)**	
No (≤150)	52.5
Yes (>150)	47.5
**High-density lipoprotein cholesterol (mg/dL) (n = 945)**	
Normal (≥40)	59.8
Low (<40)	40.2
**Low-density lipoprotein cholesterol^g^ (mg/dL) (n = 945)**	
Normal (≤160)	92.7
Elevated (>160)	7.3
**Hypertension^h^ (n = 945)**	
No	66.1
Yes	33.9
**Metabolic syndrome^i^ (n = 945)**	
No	56.0
Yes	44.0

Abbreviation: SE, standard error.

a All statistics weighted. Percentages may not reflect the expected value due to sampling weights and design-based analyses. Values expressed as percentages, unless otherwise indicated.

b “Insurance” includes both public and private coverage of any type.

c Defined as an affirmative response to, “Have you ever smoked more than 100 cigarettes in your entire life?”

d Defined as affirmative responses to 1) “Have you ever smoked more than 100 cigarettes in your entire life?” and 2) “Do you now smoke cigarettes?”

e Defined as alanine transaminase >40 u/L and/or aspartate aminotransferase >37 u/L.

f According to American Diabetes Association 2010 Diagnostic Guidelines (20).

g Calculated low-density lipoprotein cholesterol levels.

h Defined as systolic blood pressure ≥130 mm Hg or diastolic blood pressure ≥85 mm Hg or currently taking antihypertensive medication.

i According to Adult Treatment Panel III (21).

### Stratified and multiple comparisons analyses

**Figure 1:**
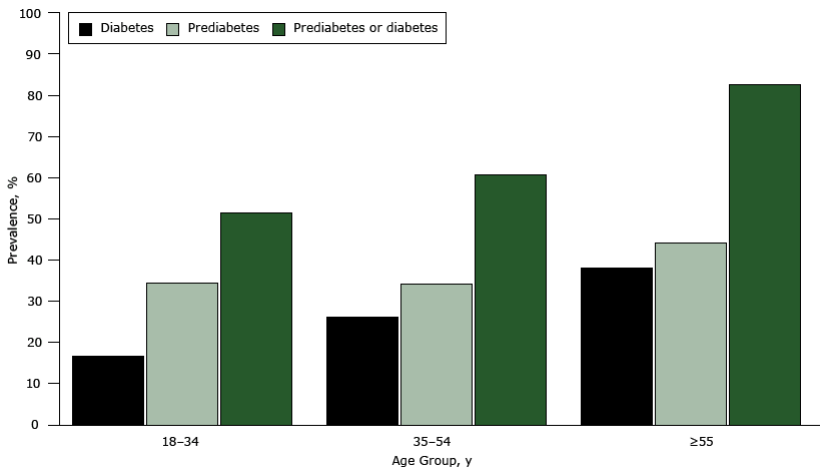
Proportion of male participants with diabetes, prediabetes, and prediabetes or diabetes, by age group, Cameron County Hispanic Cohort, 2004–2015. This figure shows that prevalence of prediabetes is above 30% across age groups and that more than 50% of men younger than 35 years in this population have either diabetes or prediabetes. **Table:** 

Obesity prevalence was similar across age groups, at approximately 50% (*P* = .83) (Table 2). Mean waist circumference (*P* = .29) and mean BMI (*P* = .19) were also similar across age groups. The proportion with diabetes was significantly associated with age group (*P* < .001). This proportion was highest in the older age group (38.2%; 95% CI, 29.3%–47.1%), although 16.9% (95% CI, 10.1%–23.8%) of the younger age group had diabetes. Additionally, 51.3% (95% CI, 42.9%–59.8%) of men in the younger group had either prediabetes or diabetes, and this prevalence was even higher in the other 2 age groups (Figure 1).

**Table 2:** Comparison of Variables, by Age Group, of Men in the Cameron County Hispanic Cohort (N = 945), 2004–2015 **a**

Categorical Variable	Age, y						*P* Value
	18–34		35–54		≥55		
	n	% (95% CI)	n	% (95% CI)	n	% (95% CI)	
Obese BMI (>30 kg/m^2^) (n = 945)	129	48.9 (40.4–57.4)	207	52.4 (45.9–58.9)	114	50.2 (40.3–60.1)	.83
Diabetes^b^ (n = 933)	39	16.9 (10.1–23.8)	106	26.3 (20.8–31.8)	116	38.2 (29.3–47.1)	<.001
Prediabetes or diabetes^b^ (n = 933)	125	51.3 (42.9–59.8)	243	60.6 (54.0–67.3)	215	82.2 (75.9–88.6)	<.001
Elevated LFTs^c^ (n = 945)	218	70.2 (62.0–78.5)	282	70.4 (64.3–76.6)	127	51.2 (41.4–61.0)	.001
Reduced HDL^d^ (n = 945)	108	41.2 (32.7–49.7)	149	35.9 (29.8–41.9)	107	44.0 (34.1–53.8)	.37
Elevated LDL^e^ (n = 933)	17	5.2 (2.3–8.2)	40	9.7 (6.3–13.1)	20	7.0 (3.2–10.8)	.19
Hypertriglyceridemia^f^ (n = 945)	104	37.5 (29.1–45.9)	226	56.9 (50.4–63.4)	137	48.1 (38.2–58.0)	.005
Hypertension^g^ (n = 945)	49	23.8 (15.3–32.3)	97	24.2 (18.8–29.6)	144	56.0 (46.5–65.6)	<.001
Metabolic syndrome^h^ (n = 945)	81	33.7 (25.2–42.1)	171	42.5 (36.1–48.8)	155	57.3 (47.4–67.3)	<.001
Smoking history^i^ (n = 945)	160	51.5 (43.1–59.9)	175	45.2 (38.6–51.7)	102	40.0 (29.6–50.3)	.20
Current smoker^j^ (n = 945)	95	35.4 (27.2–43.6)	120	29.5 (23.8–35.3)	57	19.4 (12.9–25.8)	.006
Sometimes drink (n = 945)	159	61.3 (53.5–69.1)	278	65.0 (58.7–71.3)	146	53.8 (43.9–63.8)	.15
Heavy drinking (n = 945)	35	8.2 (4.7–11.6)	15	7.5 (3.4–11.6)	17	5.7 (2.3–9.1)	.61
Insurance (n = 944)	49	19.2 (11.7–26.8)	119	30.5 (24.1–36.9)	152	61.8 (53.0–70.6)	<.001
**Continuous Variable, Mean Value**	**Age, y (95% Confidence Interval)**						***P* Value**
	**18–34**		**35–54**		**≥55**		
Waist circumference, cm (n = 945)	103.8 (100.8–106.8)		105.3 (103.5–107.1)		106.5 (104.7–108.3)		.29
BMI, kg/m^2^ (n = 945)	31.2 (29.9–32.4)		31.3 (30.5–32.2)		30.2 (29.3–31.1)		.19
SBP, mm Hg (n = 945)	116.0 (113.7–118.4)		116.8 (115.0–118.7)		125.2 (121.7–128.6)		<.001
DBP, mm Hg (n = 945)	74.1 (72.4–75.8)		75.3 (74.0–76.7)		71.8 (69.8–73.9)		.03
ALT, u/L (n = 945)	56.9 (49.5–64.3)		47.8 (45.2–50.5)		39.8 (36.5–43.0)		<.001
AST, u/L (n = 945)	45.0 (39.5–50.6)		38.0 (36.0–40.0)		33.8 (30.6–37.0)		.002
Triglycerides, mg/dL (n = 945)	182.5 (144.4–220.6)		230.9 (184.4–277.5)		195.9 (191.2–220.6)		.01
HDL, mg/dL (n = 945)	41.7 (39.4–44.0)		42.7 (40.8–44.7)		42.1 (40.4–43.9)		.71
LDL, mg/dL (n = 933)	92.2 (84.3–100.1)		108.6 (101.5–115.8)		102.2 (96.0–108.4)		<.001

Abbreviations: ALT, alanine transaminase; AST, aspartate aminotransferase; BMI, body mass index; CI, confidence interval; DBP, diastolic blood pressure; HDL, high-density lipoprotein cholesterol; LDL, low-density lipoprotein cholesterol; LFTs, liver function tests; SBP, systolic blood pressure.

a All statistics weighted. Percentages may not reflect the expected result due to sampling weights and design-based analyses.

b According to American Diabetes Association 2010 Diagnostic Guidelines (20).

c Defined as ALT >40 u/L and/or AST >37 u/L.

d Defined as <40 mg/dL.

e Defined as LDL >160 mg/dL.

f Defined as triglyceride levels >150 mg/dL.

g Defined as systolic blood pressure ≥130 mm Hg or diastolic blood pressure ≥85 mm Hg or taking antihypertensive medication.

h According to Adult Treatment Panel III (21).

i Defined as an affirmative response to, “Have you ever smoked more than 100 cigarettes in your entire life?”

j Defined as affirmative responses to 1) “Have you ever smoked more than 100 cigarettes in your entire life?” and 2) “Do you now smoke cigarettes?”

Age Group, y	Diabetes	Prediabetes	Prediabetes or Diabetes
	Prevalence, %		
18–34	16.9	34.4	51.3
35–54	26.3	34.3	60.6
≥55	38.2	44.0	82.2

**Figure 2:**
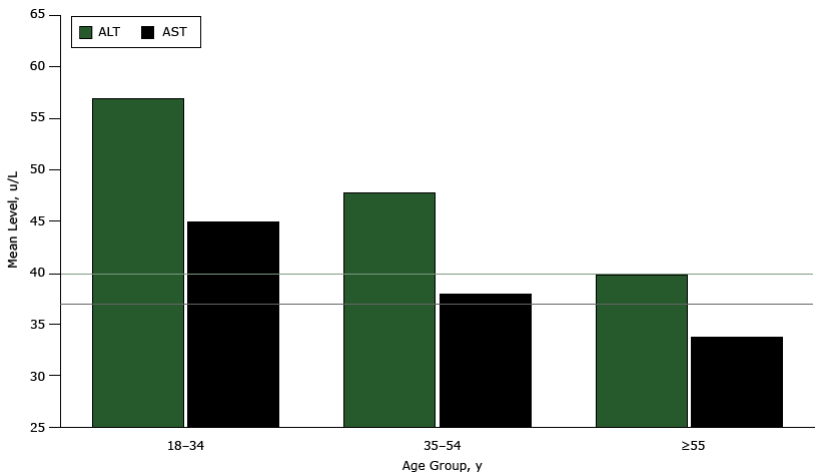
Mean levels of ALT and AST, by age group, male participants of the Cameron County Hispanic Cohort, 2004–2015. Mean levels of ALT and AST are highest in men younger than 35 and lower in older age groups. The upper limit of normal for ALT is 40 u/L, and the upper limit of normal for AST is 37 u/L (indicated by horizontal lines on graph). Abbreviations: ALT, alanine transaminase; AST, aspartate aminotransferase. **Table:** 

The proportion of men with elevated LFTs was significantly associated with age group (*P* = .001). In multiple comparisons analysis, the odds of elevated LFTs was significantly lower in the older age group than in the younger age group (OR = 0.4; 95% CI, 0.3–0.8). When examining continuous values of ALT and AST, we found differences in both mean ALT (*P* < .001) and mean AST (*P* = .002) across age groups (Figure 2). In multiple comparisons (Table 3), both ALT and AST mean levels were significantly higher in the younger group than in the older group (ALT difference: 17.2 u/L; 95% CI, 7.4–27.0; *P* < .001; AST difference: 11.2 u/L; 95% CI, 3.7–18.7; *P* = .001). AST mean levels were also significantly higher in the younger group than in the middle-aged group (difference: 7.1 u/L; 95% CI, 0.02–14.1; *P* = .05), but this finding did not remain significant when controlling for place of birth. There was an overall association between elevated triglycerides and age group (*P* = .01). Multiple comparisons indicated that the odds of elevated triglycerides was significantly higher in the middle-aged group than in the younger group (OR = 2.2; 95% CI, 1.4–3.4), as were mean levels of triglycerides (difference: −48.4 mg/dL, 95% CI, −87.1 to −9.7, *P* = .008). We observed that the younger age group had a 37.5% prevalence of elevated triglyceride levels (95% CI, 29.1%–45.9%) (Table 2). Furthermore, 33.7% of men aged 18 to 34 years (95% CI, 25.2%–42.1%) satisfied the criteria for metabolic syndrome, compared with 57.3% of men aged 55 years or older (95% CI, 47.4%–67.3%), with a significant overall association with age group (*P* < .001).

Age Group, y	Mean Level, u/L	
	ALT	AST
18–34	56.9	45.0
35–54	47.8	38.0
≥55	39.8	33.8

**Table 3:** Multiple Comparison Analysis of Variables, by Age Group, of Men in the Cameron County Hispanic Cohort (N = 945), 2004–2015

Categorical Variable	Odds Ratio^a^	
**Diabetes^b^**		
18–34	1 [Reference]	
35–54	1.8 (1.0 to 3.1)	
≥55	3.0 (1.7 to 5.6)	
**Prediabetes or diabetes**		
18–34	1 [Reference]	
35–54	1.5 (0.9 to 2.3)	
≥55	4.4 (2.6 to 7.4)	
**Elevated liver function tests^c^**		
18–34	1 [Reference]	
35–54	1.0 (0.6 to 1.6)	
≥55	0.4 (0.3 to 0.8)	
**Hypertriglyceridemia^d^**		
18–34	1 [Reference]	
35–54	2.2 (1.4 to 3.4)	
≥55	1.5 (0.9 to 2.6)	
**Hypertension^e^**		
18–34	1 [Reference]	
35–54	1.0 (0.6 to 1.8)	
≥55	4.1 (2.2 to 7.7)	
**Metabolic syndrome^f^**		
18–34	1 [Reference]	
35–54	1.5 (0.9 to 2.3)	
≥55	2.6 (4.5 to 4.6)	
**Health insurance**		
18–34	1 [Reference]	
35–54	1.8 (1.1 to 3.2)	
≥55	6.8 (3.8 to 12.3)	
**Current smoker^g^**		
18–34	1 [Reference]	
35–54	0.8 (0.5 to 1.2)	
≥55	0.4 (0.3 to 0.8)	
**Continuous Variable**	**Difference (95% Confidence Interval)**	***P* Value^h^**
**Systolic blood pressure, mmHg**		
18–34	Reference	
35–54	−0.8 (−4.5 to 2.9)	.99
≥55	−9.1 (−14.2 to −4.0)	<.001
**Diastolic blood pressure, mmHg**		
18–34	Reference	
35–54	−1.2 (−3.9 to 1.5)	.82
≥55	2.3 (−1.0 to 5.5)	.28
**Triglycerides, mg/dL**		
18–34	Reference	
35–54	−48.4 (−87.1 to −9.7)	.008
≥55	−13.4 (−58.4 to 31.6)	.99
**Low-density lipoprotein cholesterol, mg/dL**		
18–34	Reference	
35–54	−16.4 (−25.7 to −7.2)	<.001
≥55	−10.0 (−20.1 to 0.1)	.05
**Alanine transaminase levels, u/L**		
18–34	Reference	
35–54	9.1 (−0.6 to 18.9)	.08
≥55	17.2 (7.4 to 27.0)	<.001
**Aspartate aminotransferase levels, u/L**		
18–34	Reference	
35–54	7.1 (0.02 to 14.1)^i^	.05^i^
≥55	11.2 (3.7 to 18.7)	.001

a Survey-weighted odds ratio generated from logistic regression.

b According to American Diabetes Association 2010 Diagnostic Guidelines (20).

c Defined as alanine transaminase >40 u/L and/or aspartate aminotransferase >37 u/L.

d Defined as triglyceride levels >150 mg/dL.

e Defined as systolic blood pressure ≥130 mm Hg or diastolic blood pressure ≥85 mm Hg or taking antihypertensive medication.

f According to Adult Treatment Panel III (21).

g Defined as affirmative responses to 1) “Have you ever smoked more than 100 cigarettes in your entire life” and 2) “Do you now smoke cigarettes?”

h Bonferroni adjusted *P* values for multiple pairwise comparisons.

i Nonsignificant after controlling for place of birth.

The proportion of men with health insurance was highest in the older age group (61.8%; 95% CI, 53.0%–70.6%), and lowest in the younger age group (19.2%; 95% CI, 11.7%–26.8%), with an overall significant association (*P* < .001) between age group and insurance (Table 2). Having health insurance was also significantly associated with being born in the United States (OR = 2.1; 95% CI, 1.4–3.2; data not shown), but both age group and place of birth remained independently associated with insurance status in logistic regression (data not shown).

Rates of current smoking were highest in the younger age group (35.4%; 95% CI, 27.2%–43.6%) and lowest in the older age group (19.4%; 95% CI, 12.9%–25.8%), with an overall significant association with age (*P* = .006). Multiple comparisons indicated that current smoking prevalence was significantly higher in the younger group than in the older group (OR [older vs younger] = 0.4; 95% CI, 0.2–0.7) but similar to the middle group. Overall history of smoking (past or present) and drinking behavior had no significant associations with age group.

## Discussion

This article is among the few population-based studies of the health of Mexican American men, and it allows for evidence-based risk stratification by age in the Mexican American population. Before this study, little was known about the health needs of Mexican American men in the southern Texas–Mexico border region. Our results show strikingly adverse metabolic and behavioral outcomes in men younger than 35 years. Poor metabolic health (eg, dyslipidemia, elevated blood pressure, obesity, and prediabetes) appears to extend into the 35 to 54 years age group. Variables that tend to be associated with older age — such as hypertension, diabetes, and metabolic syndrome — were also associated with older age in this population. Mean BMI and mean waist circumference were uniform but high across all age groups. Although mounting evidence suggests that metabolic health, as opposed to obesity, is a more important indicator of cardiovascular risk (16,23,24), obesity is an important predictor of several health outcomes. In this population, obesity begins in adolescence (25) and persists through middle and older age.

A relevant finding from this study was the significant burden of obesity, prediabetes, and diabetes in the younger age group. Outcomes such as obesity tend to peak during middle age in men (26), but we found a 48.9% prevalence of obesity in younger men. For comparison, the HCHS/SOL study found a 36.8% prevalence of obesity in 2,337 Mexican American men in their nationwide survey (10); our findings suggest a higher burden of obesity in Mexican American men residing on the Texas–Mexico border than in Mexican American men nationwide. We found a 34.3% prevalence of prediabetes and 16.9% prevalence of diabetes in the younger group. Although high rates of diabetes have been documented in this population (15,27), our findings indicate a high prevalence of diabetes in young men, which has not been widely addressed in the literature. Nationwide, the overall prevalence of diabetes among Mexican American men is 18.7% (28), so the prevalence of diabetes in men younger than 35 in this population is nearly as high as the nationwide average for all ages. Employing widely used cut-offs for elevated ALT and AST, we found a 70.2% prevalence of elevated LFTs in the younger group, which was similar to the prevalence for the middle-aged group and significantly higher than that for the older group. Research indicates that ALT levels may decrease slightly with age (29), but our findings were nonetheless remarkable. Given the documented high rates of nonalcoholic fatty liver disease in this cohort (30), the authors believe that this association is real and that the drivers of elevated liver enzymes in young Mexican American men warrant further study.

The younger group fares worse than the older groups in 2 other important measures: lack of health insurance and high rates of current smoking. We found that less than 20% of men younger than 35 had any health care coverage. Limited resources exist for uninsured, and especially undocumented, men to obtain affordable health care, so preventive care may not be sought by this young population. Among men in the younger age group, 51.5% had a history of smoking and 35.4% identified as current smokers. The percentage of young men with any history of smoking was higher (though not significantly) than the percentage of older men with a history of smoking (51.5% vs 40.0%). This finding suggests that smoking initiation across generations is consistent. Both the rate of current smoking among younger men in the CCHC (35.4%) and rate of current smoking among all men in the CCHC (28.4%) appear to be higher than the overall rate of current smoking in US Hispanic men (17.3%) (31).

Although poor health outcomes were not restricted to the younger group, the findings in this group provoke the greatest concern from a prevention perspective. The high prevalence of poor metabolic health outcomes in Mexican Americans is now well documented (14,17,26), but the high prevalence of poor metabolic health outcomes in young men has not yet been adequately studied. Given that more than 60% of the overall male population in this region lacks health insurance and that men in general are less likely to exhibit health-seeking behavior than women (4), these data support the need for aggressive chronic disease intervention programs for young Mexican American men.

There were several limitations to this study. The data we used were cross-sectional and do not provide insight into changes in health over time or causality. Only longitudinal data will allow us to determine whether the differences in the age groups are cohort effects or whether we may see premature death in metabolically unhealthy young men, or both. Additionally, many participants declined to provide income information. Despite these limitations, we contribute to the characterization of Mexican American men’s health, affirming the importance of stratified analyses of health among Hispanic men of distinct ages and ethno-regional subgroups.

There are active obesity and diabetes prevention programs in South Texas, but we suspect that men are missed by these efforts. For example, the Coordinated Approach to Child Health (CATCH) targets obesity in children and families, and “Salud y Vida” aims to prevent diabetes in people with prediabetes. However, less than 30% of participants in “Salud y Vida” are men (M. Zolezzi, University of Texas School of Public Health, written communication, 2015). Men rarely attend free exercise classes offered by the University of Texas School of Public Health (A. Davé, University of Texas School of Public Health, written communication, 2016). The literature corroborates these findings, suggesting that interventions should be consciously tailored toward men (32). Additionally, we suspect that vastly different approaches are needed for each age group. We believe our findings will contribute to a re-evaluation of intervention programs in the region and shape new interventions targeting men at highest risk. Our data also suggest that men in South Texas would benefit from culturally appropriate smoking cessation programs. Lower smoking rates would consequently reduce overall cardiovascular risk. By prioritizing younger men for primary prevention, we can reduce poor health behaviors (eg, poor diet, sedentary lifestyle, tobacco use) that begin in early adulthood or even before and mitigate the burden of more severe disease (eg, cardiovascular disease, diabetes, cancer) later in life.
